# The role of selenium in depression: a systematic review and meta-analysis of human observational and interventional studies

**DOI:** 10.1038/s41598-022-05078-1

**Published:** 2022-01-20

**Authors:** Sana Sadat Sajjadi, Sahar Foshati, Sajjad Haddadian-Khouzani, Mohammad Hossein Rouhani

**Affiliations:** 1 Food Security Research Center, Department of Community Nutrition, School of Nutrition and Food Science, Isfahan University of Medical Sciences, Isfahan, Iran; 2 Food Security Research Center, Department of Clinical Nutrition, School of Nutrition and Food Science, Isfahan University of Medical Sciences, Isfahan, Iran

**Keywords:** Medical research, Neurology

## Abstract

The results of human studies are inconsistent regarding selenium and depressive disorders. Therefore, we aimed to conduct a systematic review and meta-analysis of observational and interventional studies and provided an overview of the role of selenium in depression. Three databases including Medline, Scopus, and Web of Science were searched on June 30, 2020 and updated on April 12, 2021. Also, we searched in electronical databases of WHO Global Index Medicus and ClinicalTrials.gov. No time or language restrictions were used for the search. A random effects model was used to pool effect sizes. In total, 20 studies were included in the systematic review, and 15 studies were included in the meta-analysis. There were no significant differences in serum selenium levels between patients with depression and healthy subjects (WMD: 2.12 mg/L; 95% CI: − 0.11, 4.36; *I*^2^ = 98.0%, *P* < 0.001). Also, no significant correlation was found between serum levels of selenium and depression scores (r: − 0.12; 95% CI: − 0.33, 0.08; *I*^2^ = 73.5%, *P* = 0.010). Nevertheless, there was a significant negative association between high selenium intake and the risk of postpartum depression (OR: 0.97; 95% CI: 0.95, 0.99; *I*^2^ = 0.0%, *P* = 0.507). In addition, selenium supplementation significantly reduced depressive symptoms (WMD: − 0.37; 95% CI: − 0.56, − 0.18; *I*^2^ = 0.0%, *P* = 0.959). Taken these results together, selenium seems to have a protective role against postpartum depression and can be considered as a beneficial adjuvant therapy in patients with depression. Further studies are necessary to draw definitive conclusions.

## Introduction

Depression is identified as a disabling mental illness, which can substantially impair quality of life^1,2^. According to the report of World Health Organization in 2018, more than 264 million people are affected by depression worldwide^3^. The rate of depression dramatically increased from 172 to 258 million since 1990 to 2017, showing a 50% increase^4^. Patients with depression may experience fatigue, sleep disturbance, loss of memory and concentration, poor appetite, loss of work motivation, and low self-confidence during their lives^5,6^. Also, untreated depression can lead to serious social problems and even suicide^7^.

It appears that nutrition plays a critical role in mental health^8^. For instance, several studies have supported the favorable effects of magnesium, vitamin D, B-vitamins, and omega-3 fatty acids on mood disorders^9^. Moreover, recent evidence has revealed the benefits of using trace elements in the prevention and treatment of depression^10^. Among trace elements, selenium may be of great importance in the management of depression due to its antioxidant, anti-inflammatory, immunomodulatory, and neuroprotective properties^11,12^. In addition to depression, selenium deficiency may be associated with many other diseases such as type 2 diabetes mellitus, cardiovascular disease, kidney diseases, infertility, and cognitive decline^13,14^.

The results of observational and interventional studies are inconsistent regarding the role of selenium in depression^15–22^. Several studies reported a significant negative relationship between dietary or serum levels of selenium and the risk of depression^15–17^. In contrast, some studies did not find such a relationship^18,19^. Even, a narrative review generally reported that there is an unclear relationship between selenium and depression^23^. Furthermore, selenium supplementation showed a positive effect on depression symptoms in some clinical trials^20,21^ but a neutral effect in others^22^. Since no comprehensive review article has yet been published on this controversial topic, we aimed to conduct a systematic review and meta-analysis of human observational and interventional studies and provide an overview of the role of selenium in depressive disorders.

## Methods

### Search strategy

This study was conducted according to the guidelines of Meta-Analysis of Observational Studies in Epidemiology (MOOSE) for observational studies^24^ and Preferred Reporting Items for Systematic Reviews and Meta-Analyses (PRISMA) for interventional studies^25^. A systematic electronic search was performed to identify all publications on selenium and depression. Three databases including Medline (via PubMed), Scopus, and Web of Science were searched on June 30, 2020 and updated on April 12, 2021. Also, we searched in electronical databases of WHO Global Index Medicus and ClinicalTrials.gov. No time or language restrictions were used for the search. The following Medical Subject Headings (MeSH) and non-MeSH terms were used to identify potential studies: (“depression” OR “depressions” OR “depressive disorder” OR “depressive disorders” OR “depressive” OR “depressed”) AND (“selenium” OR “selenite” OR “selenite” OR “seleno” OR “Se”).

### Eligibility criteria

The retrieved articles were included in the present study if they met the following criteria: (1) had an observational (cross-sectional, case–control, and cohort) or an interventional (randomized controlled trial) design, (2) conducted on humans, (3) investigated the association between dietary or supplementary intake or serum levels of selenium and depression, (4) compared dietary or supplementary intake or serum levels of selenium between patients with depression and healthy controls, and (5) assessed the effect of selenium supplementation or selenium rich diet on depression. The exclusion criteria were: (1) reviews, books, case reports, conference papers, letters to the editor, and animal or in vitro studies, (2) studies which failed to assess selenium, (3) studies which administered selenium in combination with other components, (4) studies which measured nail or hair selenium, and (5) studies reported duplicate data, (6) studies which assessed other outcomes other than depression, (7) protocol study, and (8) studies which failed to assess the association between selenium and depression.

### Data extraction and quality assessment

The following characteristics were collected from the included publications: the first author’s last name, year of publication, country where the study was conducted, sample size, gender and mean or median age of participants, study design, type of depressive disorder, assessment tool of depression, adjusted covariates, main results, values of selenium intake or levels of serum or plasma selenium, serum selenium values at baseline and after-treatment, methodologies of selenium measurement. The quality assessment of observational studies was performed using the Newcastle–Ottawa Scale (NOS)^26^, and the quality of interventional studies was evaluated using the Cochrane Collaboration Risk of Bias Tool (CCRBT)^27^.

### Data synthesis and analysis

To improve normal distribution, correlation coefficients between serum selenium levels and depression scores were converted to z-values using Fisher's r-to-z transformation. Subsequently, following formula was used to converted back to r-values when effect sizes were calculated: ES (z) = ½ ln [(1 + r)/(1 − r)]^28^. We converted standard errors (SE) to standard deviations (SD) using the formula SD = SE × √N. To calculate SD from 95% confidence interval, following formula was used: SD = √N × (upper limit − lower limit) ÷ 3.92. A random-effects model was used to calculate pooled effect size to compare serum selenium levels between depressive patients and healthy controls^29^. We used the random-effects model because inter-study heterogeneity was high. The random-effects model should be used for pooling heterogeneous studies^30^. Similar method was applied to compare change in depression scores between selenium supplementation and control groups. Since included clinical trials used different tools to assess depression score, pooled effect was calculated via Hedges' g^31^. Log-transformed odds ratios of depression across different categories of selenium intake were also applied to calculate overall effect sizes. Overall effect sizes were reported as odds ratio (OR), weighted mean difference (WMD) and correlation coefficient (r). I‐squared (*I*^2^) statistic was reported as an indicator of between-study heterogeneity. To detect the potential sources of heterogeneity, a subgroup analysis was applied when a significant between-study heterogeneity was observed. Sensitivity analysis was performed as a complementary analysis to assess robustness of results. Begg's rank correlation test and Egger's linear regression test were used to test publication bias. The potential effect of publication bias was assessed using trim-and-fill analysis. All statistical analyses were performed using Stata software (version 11.2, Stata Corporation, College Station, Texas, USA); additionally, analyses were two-tailed, and statistical significance was set at *P* < 0.05.

## Results

### Study selection process

**Figure 1:**
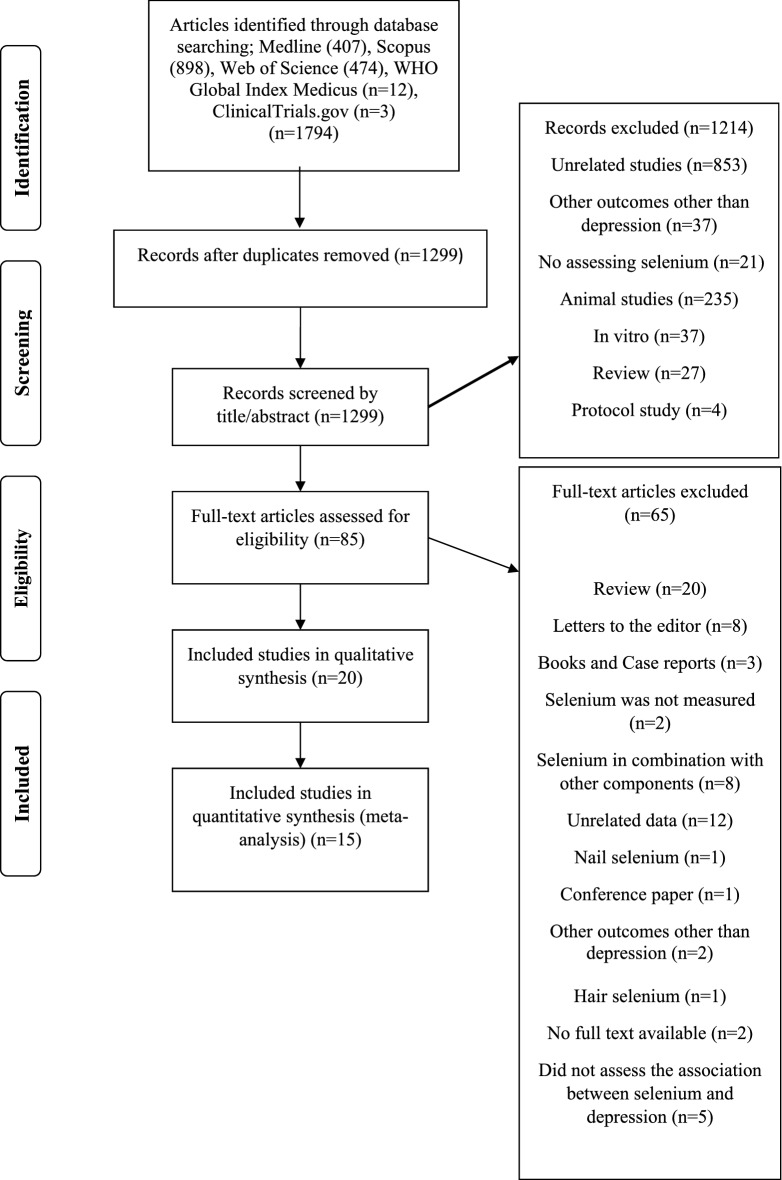
Flow diagram of the study selection process.

Initially, 1794 published articles were identified from the electronic databases (Fig. 1). After removing 495 duplicates, 1299 records were assessed for eligibility, and 1214 studies were excluded based on screening title and abstract (unrelated studies (n = 853), evaluation other outcomes other than depression (n = 37), selenium intake/concentration was not reported (n = 21), animal studies (n = 235), in vitro studies (n = 37), review articles (n = 27) and protocol studies (n = 4). After screening full-text of the records, 65 studies were excluded due to the following reasons: review articles (n = 20), letters to the editor (n = 8), books and case reports (n = 3), failure to assess selenium (n = 2), administration of selenium in combination with other components (n = 8), unrelated data (n = 12), measurement of nail selenium levels (n = 1), conference papers (n = 1), evaluation other outcomes other than depression (n = 2), measurement of hair selenium levels (n = 1) full text of articles were not available at databases or journal website (n = 2) and the association between selenium and depression was not assessed (n = 5). Finally, 20 studies^15–22,32–43^were included in the systematic review.

### Systematic review

Characteristics of studies eligible for the systematic review are summarized in Table 1. These studies were published between 2003 and 2020. Among twenty studies, two were conducted in New Zealand^15,34^, four in Iran^16,18,20,41^, three in the US^22,37,38^, one in the UK^21^, three in Spain^17,35,36^, one in Bangladesh^40^, one in Canada^33^, one in Australia^43^, one in Pakistan^39^, one in Poland^19^, one study in Columbia^42^, and one study in.

**Table 1:** Overview of the studies included in the systematic review.

First author (year)	Country	Sample size (male/female)	Age (mean ± SD, median (IQR), year)	Design	Reported data	Type of depression	Depression assessment tool	Results	Adjusted variables
Amini (2019)	Iran	163 (0/163)	27.79 ± 6.1	Case–control	Risk of depression, Mean of dietary selenium	PPD	EPDS	A more selenium intake was associated with an reduced risk of depression	Energy intake and BMI
Banikazemi (2016)	Iran	7172 (2725/4447)	48.55 ± 7.4	Cross-sectional	Risk of depression	NR	BDI	Selenium intake was negatively associated with the relative risk of a high depression score	Energy intake
Conner (2015)	New Zealand	978 (357/621)	19.6 ± 1.6	Cross-sectional	Mean of depression score	NR	CESD	A negative association between serum selenium and risk of depression	Age, gender, ethnicity, BMI, and mean weekly alcohol intake
Ekramzadeh (2015)	Iran	150 (17/133)	47.23 ± 13.6	Cross-sectional	Correlation between serum selenium and depression score, Mean of serum selenium	NR	BDI	No significant association between depression score and serum selenium	Age, sex, marriage, job, and education level
Ghimire (2019)	US	7725 (3723/4002)	46.4 (32.5–59.7)	Cross-sectional	Risk of depression (serum and dietary)	NR	PHQ-9	An inverse association between dietary selenium and depression, No significant association between serum selenium and depression	Age, sex, race, ethnicity, marital status, educational status, family poverty income ratio, BMI, smoking, alcohol use, physical activity, use of dietary supplements, diabetes, kidney disease, cancer, heart disease, and energy intake
Gosney (2008)	UK	59 (NR)	82 (NR)	Randomized controlled trial	Correlation between serum selenium and depression score, Effect of selenium supplementation on depression score	NR	MADRS	A significant negative relationship between serum selenium and depression, Significant reduction in depression score in active group	NR
Ibarra (2015)	Spain	77 (18/59)	50.46 ± 11.6	Randomized controlled trial	Correlation between serum selenium and depression score	MDD	HDRS-17 BDI	Active group had a better outcome of depressive symptoms, An inverse association between serum selenium and depression	NR
Islam (2018)	Bangladesh	495 (192/303)	33.29 ± 0.6	Case–control	Mean of serum selenium in healthy and depressed subjects	MDD	SCID-5	MDD patients had lower levels of selenium	NR
Jin (2020)	New Zealand	87 (0/87)	31.5 ± 4.2	Cohort	Median of serum selenium	PPD	EPDS	No significant association between plasma selenium values and prevalence of depression	NR
Leung (2013)	Canada	475 (0/475)	31.4	Cohort	Risk of depression	PPD	EPDS	Supplementary selenium intake was negatively associated with the risk of depression	NR
Li (2018)	US	14,834 (7399/ 7435)	24.99	Cross-sectional	Risk of depression	NR	PHQ-9	Total selenium intake was negatively associated with depression	BMI, race, educational level, smoking status, family income, work activity, recreational activity, hypertension, diabetes, energy intake, age, and gender
Mokhber (2011)	Iran	85 (0/85)	21.61 ± 2.9	Randomized controlled trial	Effect of selenium supplementation on depression score	PPD	EPDS	Selenium group had lower mean EPDS score	NR
Pasco (2012)	Australia	316 (0/316)	54.5	Nested case–control	Risk of depression	MDD	SCID-I	A low selenium intake was associated with an increased risk of de novo MDD	Age, socioeconomic status, smoking, alcohol use, and physical activity
Perez-Cornago (2015)	Spain	84 (47/37)	49.4 ± 2.7	Cross-sectional	Mean of dietary selenium	NR	BDI	Intake of more selenium was associated with better mood	Sex, age, and energy intake
Samad (2019)	Pakistan	96 (13/83)	50	Case–control	Mean of serum selenium in healthy and depressed subjects	NR	HDRS-17	Depression was associated with selenium deficiency	NR
Sánchez‑Villegas (2018)	Spain	13,983 (5880/8103)	38.2 ± 11.9	Cohort	Risk of depression	NR	SCID-I	Inadequate selenium intake was related to increased risk of depression	Sex, age, physical activity, energy intake, alcohol intake, BMI, special diets, smoking, and prevalence of diseases such as cardiovascular disease, hypertension, and type 2 diabetes
Shor-Posner (2003)	Miami	63 (32/31)	40.0 ± 6.4	Randomized controlled trial	Effect of selenium supplementation on depression score	NR	BDI	No significant change in the prevalence of depression	NR
Singh (2017)	Columbia	108 (0/108)	18.0 ± 1.2	Cross-sectional	Correlation between selenium intake and depression score	Pregnant depression	RADS	No significant association between selenium intake and depressive symptoms	Energy intake
Wieder-Huszla (2020)	Poland	102 (0/102)	56.69 ± 6.0	Cross-sectional	Correlation between serum selenium and depression score	Postmenopausal depression	BDI	No significant association between depression score and serum selenium	NR
Tatt (2019)	Malaysia	112 (56/56)	71.4 ± 7.0	Cross-sectional	Correlation between selenium intake and depression score	NR	GDS-15	No significant association between GDS score and selenium intake, but a negative association between selenium intake and GDS score in males	NR

NR: Not reported, BMI: Body mass index, MDD: Major depressive disorder, PPD: Postpartum depression, EPDS: Edinburgh Postnatal Depression Scale, CESD: Center for Epidemiological Studies–Depression, BDI: Beck Depression Inventory, MADRS: Montgomery-Asberg Depression Rating Scale, HDRS-17: 17-item Hamilton Depression Rating Scale, PHQ-9: 9-item Patient Health Questionnaire, SCID-5: Structured Clinical Interview for DSM-5, SCID-I: Structured Clinical Interview for DSM-IV Axis I Disorders, RADS: Reynolds Adolescent Depression Scale. GDS-15:15-items Chinese Geriatric Depression Scale.

Malaysia^32^. Four studies were randomized controlled trials^20–22,36^, nine studies used a cross-sectional design^15,18,19,32,35,37,38,41,42^, four were case–control studies^16,39,40,43^, and design of three studies were prospective cohort^17,33,34^. In total, 47,164 participants were enrolled in this systematic review. The age of participants ranged from 18.0 ± 1.2 to 82 years old. Twelve studies included both men and women^15,17,18,22,32,35–41^, one study did not report the gender of participants^21^, and seven studies enrolled women only^16,19,20,33,34,42,43^. Confounding variables were adjusted in half of included studies^15–18,35,37,38,41–43^. Serum selenium concentrations were measured in four cross-sectionals^15,18,19,38^, two case-controls^39,40^, and one prospective cohort^34^. Selenium intake levels were used in two case-controls^16,43^, six cross-sectionals^32,35,37,38,41,42^, and two prospective cohort studies^17,33^. Only nine studies specified the type of depression including postpartum depression (n = 4)^16,20,33,34^, major depressive disorder (n = 3)^36,40,43^, pregnant depression (n = 1)^42^, and postmenopausal depression (n = 1)^19^. The Beck Depression Inventory^18,19,22,35,36,41^ as well as Edinburgh Postnatal Depression Scale^16,20,33,34^ were the mostly used depression assessment tools in the included studies. Serum concentration, dietary or supplementary intake of selenium, and method used to measure dietary/serum selenium are reported in Supplementary Table 1. Five studies used 24-h dietary recalls to evaluate dietary or supplementary selenium intakes^32,37,38,41,42^. On the other hand, five studies used food-frequency questionnaires, 48-h weighted food records or a supplement intake questionnaire^16,17,33,35,43^. Most studies reported mean and median dietary or supplementary intake of selenium. One study reported dietary selenium as quintiles^38^. Serum concentration of selenium was reported as mean, median or tertile.

Two cross-sectional studies found no significant correlation between selenium intake and depressive symptoms^32,42^, whereas in one of the studies, there was a significant inverse correlation between selenium intake and depressive symptoms in males^32^. Also, two cross-sectional studies found no significant correlation between serum selenium and depression scores^18,19^. In addition, one cross-sectional study revealed a negative association between serum selenium levels and the risk of depression^15^. Furthermore, two case–control studies reported lower levels of selenium in depressive subjects compared to healthy controls^39,40^. Moreover, three cross-sectionals^37,38,41^, two case-controls^16,43^, and two cohorts^17,33^ reported a significant negative association between selenium intake and the risk of depression.

Two randomized controlled trials reported correlation coefficients. They found a significant inverse relationship between serum selenium levels and depression symptoms^21,36^. All interventional studies used selenium supplements except for one study that assessed the effect of selenium rich diet on depression symptoms^36^. The dose of selenium supplementation was varied from 100 to 200 μg^20–22^. A beneficial effect of selenium on depressive symptoms was reported in three studies^20,21,36^. However, one clinical trial found no significant effect of selenium on depression scores^22^.

### Quality assessment of studies

The results of the CCRBT showed that all included randomized controlled trials had high quality (Table 2). According to the NOS, all case–control and prospective cohort studies obtained ≤ 4 stars, i.e., low quality scores (Tables 3, 4). Similarly, the quality of all cross-sectional studies was low except for Ghimire^38^ and Li^37^ that respectively received good and excellent quality (Table 5).

**Table 2:** Quality assessment of the included randomized controlled trials according to the Cochrane Collaboration Risk of Bias Tool.

First author (year)	Random sequence generation (selection bias)	Allocation concealment (selection bias)	Blinding of participants and personnel (performance bias)	Blinding of outcome assessment (detection bias)	Incomplete outcome data (attrition bias)	Selective reporting (reporting bias)	Other sources of bias	Score
Shor-Posner (2003)	+	+	+	+	+	+	+	High
Mokhber (2011)	+	+	+	+	–	+	+	High
Ibarra (2015)	+	+	?	?	+	+	+	High
Gosney (2008)	+	+	+	+	+	+	+	High

Symbols: +, low risk of bias; ?, unclear risk of bias; –, high risk of bias.

**Table 3:** Quality assessment of the included case–control studies according to the Newcastle–Ottawa Scale.

First author (year)	Adequate definition of cases	Representativeness of cases	Selection of controls	Definition of controls	Control for important factors or additional factors	Exposure assessment	Same method of ascertainment for cases and controls	Non-response rate	Total quality score
Amini (2019)	–	–	–	–	*	–	*	*	3
Islam (2018)	–	–	*	*	–	*	*	–	4
Pasco (2012)	–	–	–	*	**	–	*	–	4
Samad (2019)	–	–	–	–	–	*	–	–	1

**Table 4:** Quality assessment of the included cohort studies according to the Newcastle–Ottawa Scale.

First author (year)	Representativeness of the exposed cohort	Selection of the non-exposed cohort	Ascertainment of exposure	Outcome not present at start of study	Comparability of cohorts	Assessment of outcome	Follow-up long enough for outcomes to occur	Adequacy of follow-up of cohorts	Total quality score
Jin (2020)	–	*	*	–	–	–	–	–	2
Leung (2013)	–	*	–	–	–	–	–	–	1
Sánchez‑Villegas (2018)	–	*	–	*	*	–	*	–	4

**Table 5:** Quality assessment of the included cross-sectional studies according to the Newcastle–Ottawa Scale.

First author (year)	Representativeness of the sample	Sample size	Non-respondents	Ascertainment of the exposure	Comparability of outcome groups	Assessment of outcome	Statistical test	Total score
Banikazemi (2016)	–	–	–	–	–	*	–	1
Conner (2015)	–	–	–	*	*	*	*	4
Ekramzadeh (2015)	–	–	–	*	–	*	*	3
Ghimire (2019)	*	–	–	*	**	*	*	6
Li (2018)	*	*	–	*	**	*	*	7
Perez-Cornago (2015)	–	–	–	*	–	*	*	3
Singh (2017)	–	–	*	*	–	*	*	4
Wieder-Huszla (2020)	–	–	–	*	–	*	*	3
Tatt (2019)	*	–	*	*	–	*	*	5

### Meta-analysis

From 20 studies included in systematic review, five studies were not selected for meta-analysis^15,32,34,35,42^. Two studies not included to meta-analysis reported the correlation coefficient between dietary intake of selenium and depression^32,42^. We could not pool these two studies because the score of depression was derived from different depression assessment instruments. One study was not included to meta-analysis because it reported median depression score across tertiles of plasma selenium concentration^34^. A reported selenium intake across tertiles of mood thermometer^35^. Since similar report was not found in other studies, we did not include this study to meta-analysis. Another study not selected for meta-analysis reported regression coefficient^15^. Therefore, a quantitative analysis was performed on 15 studies including 45,795 participants^16–22,33,36–41,43^.

**Figure 2:**
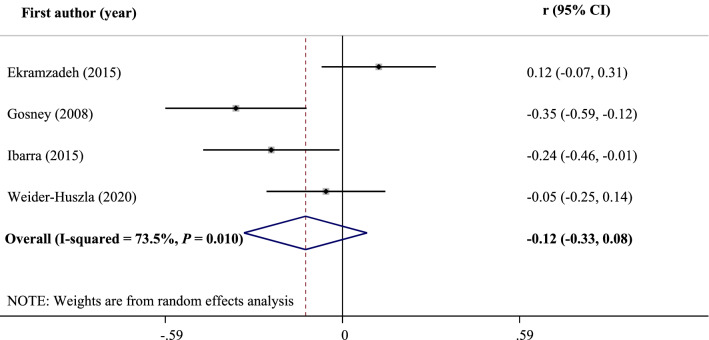
Forest plot of the correlation between serum selenium levels and depression scores.

Correlation between serum selenium levels and depression scores was assessed in four studies. The meta-analysis showed no significant correlation between serum levels of selenium and depression scores (r: − 0.12; 95% CI: − 0.33, 0.08) (Fig. 2). Although a significant between-study heterogeneity was found (*I*^2^ = 73.5%, *P* = 0.010), we could not run a subgroup analysis due to the insufficient number of studies. Moreover, there was an evidence of significant publication bias using Egger's (*P* = 0.029) and Begg's (*P* = 0.042) tests. Notwithstanding, trim-and-fill analysis indicated that no trimming could be performed and the data remained unchanged.

**Figure 3:**
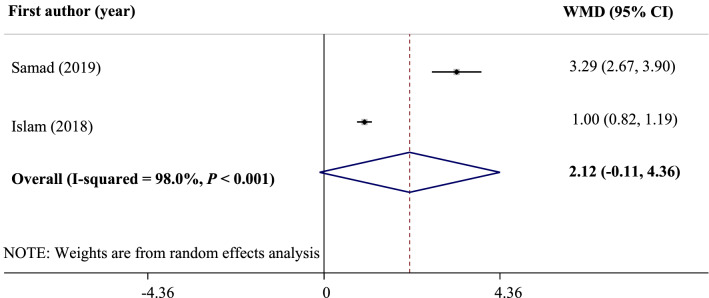
Forest plot of the comparison of serum selenium levels between depressive patients and healthy controls.

Comparison of serum selenium levels between depressive patients and healthy controls was reported in two studies. As shown in Fig. 3, the pooled results revealed that there were no significant differences in serum selenium concentrations between patients with depression and healthy subjects (WMD: 2.12 mg/L; 95% CI: − 0.11, 4.36). There was a significant heterogeneity between studies (*I*^2^ = 98.0%, *P* < 0.001). However, we could not run a subgroup analysis because of the insufficient number of studies. Moreover, the result did not show any evidence of publication bias using Begg's test (*P* = 0.31). Egger's test was not run for this section due to the insufficient number of studies.

**Figure 4:**
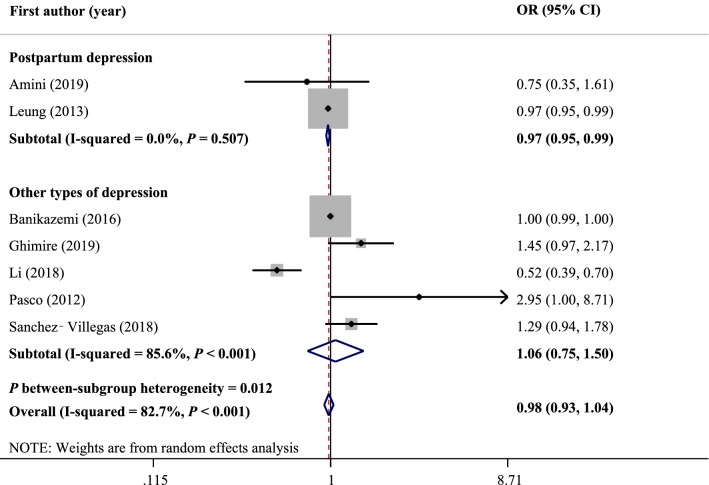
Forest plot of the association between selenium intake and the risk ratio of depression stratified by the type of depression.

Association between selenium intake and the risk of depression was reported in seven studies. The pooled risk of depression in the highest compared with the lowest categories of selenium intake was 0.98 with 95% CI of 0.93 to 1.04. A significantly high heterogeneity was observed between studies (*I*^2^ = 82.7%, *P* < 0.001). Therefore, we subgrouped studies based on the type of depression (postpartum or other types of depression) (Fig. 4). There was a significant association between selenium intake and the risk of postpartum depression (OR: 0.97; 95% CI: 0.95, 0.99; *I*^2^ = 0.0%, *P* = 0.507). Nevertheless, no significant association was found between selenium intake and the risk of other types of depression (OR: 1.06; 95% CI: 0.75, 1.50; *I*^2^ = 85.6%, *P* < 0.001). Between-subgroup heterogeneity was also high for the type of depression (*P* = 0.012). Moreover, we did not find any evidence of publication bias using Egger's (*P* = 0.65) and Begg's (*P* = 0.80) tests.

**Figure 5:**
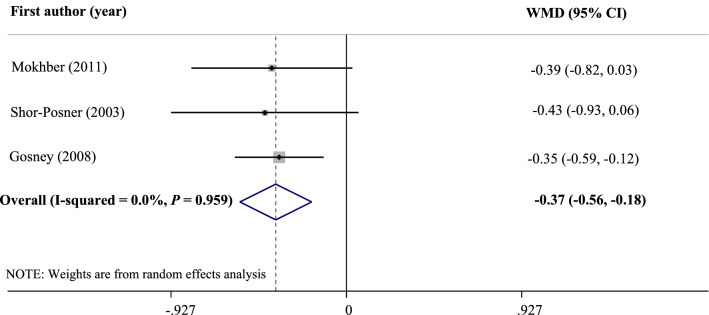
Forest plot of the effect of selenium supplementation on depression scores.

The Effect of selenium supplementation on depression scores was examined in three studies. The effect of supplementation with selenium on depression scores is shown in Fig. 5. The meta-analysis indicated a significant reduction in depression symptoms following selenium supplementation compared with placebo (WMD: − 0.37; 95% CI: − 0.56, − 0.18). There was no significant heterogeneity between studies (*I*^2^ = 0.0%, *P* = 0.959). Moreover, no evidence of publication bias was found using Egger's (*P* = 0.11) and Begg's (*P* = 0.12) tests.

### Sensitivity analysis

The sequential exclusion of each study from the pooled analysis did not significantly change the overall effect sizes except for the correlation between serum selenium concentrations and depression scores. This was significantly altered by excluding the study of Ekramzadeh et al. (r: − 0.20; 95% CI: − 0.381, − 0.031). In addition, the sequential removal of each study from the pooled analysis did not eliminate the heterogeneity except for the association between selenium intake and the relative risk of depression.

## Discussion

This meta-analysis revealed that there was no significant correlation between serum selenium levels and depression scores. In addition, no significant differences were observed between depressive and healthy subjects in serum selenium concentrations. In contrast, a significant inverse association was found between selenium intake and the risk of postpartum depression. Moreover, the meta-analysis of randomized controlled trials indicated a significant reduction in depression symptoms after selenium supplementation compared with placebo. To the best of our knowledge, the present study is the first systematic review and meta-analysis of human observational and interventional studies that comprehensively investigated the role of selenium in depressive disorders. Prior to this study, three systematic reviews suggested that nutrients such as selenium may be protective against postpartum depression. Nevertheless, these studies only focused on perinatal depression, not other types of depression. Moreover, they did not run a meta-analysis^44–46^.

The findings of this meta-analysis did not show any significant correlation between serum selenium concentrations and depression scores. In contrast, one study reported that there was a significant direct association between high dietary selenium intake and mood improvement^47^. According to the previous studies, serum selenium levels could not estimate the absolute intake of selenium^48^. In fact, some factors including demographic variables and health status may influence serum selenium concentrations^15^. Among the studies included in the present meta-analysis, confounding variables were adjusted in only one study^18^. Moreover, it seems that brain function is impaired by long-term (not short-term) exposure to low serum selenium levels^49^. In spite of this fact, the included studies reported no data regarding the duration of selenium deficiency. These reasons may explain the non-significant association between serum selenium and depression symptoms in our meta-analysis. Nevertheless, it is noteworthy that removal of the study by Ekramzadeh et al. significantly changed this result and brought about a significant negative correlation between serum concentrations of selenium and depression scores. Ekramzadeh et al. investigated the relationship of serum selenium with depression in hemodialysis subjects. They measured serum levels of selenium before the beginning of the hemodialysis session and adjusted multiple confounding factors, unlike other three included studies^18^.

In this study, no significant association was observed between selenium intake and the risk of depression. The included observational studies estimated selenium intake from foods as well as nutritional supplements. Therefore, it is possible that their results were confounded by the bioavailability of dietary or supplementary selenium. Cumulative evidence has proposed that selenium bioavailability is affected by the chemical form of selenium (organic or inorganic). Organic selenium is more bioavailable than inorganic selenium and also retains in tissues more^50^. Similarly, the effectiveness of inorganic supplements of selenium has been reported to be less than that of organic supplements^51^. Moreover, components such as heavy metals, fiber, lipids, dietary sulfur, and oxalate can have antagonistic effects on the bioavailability of dietary selenium^52,53^. Furthermore, selenium methionine and selenium cysteine were decreased during cooking processes^54^. Unfortunately, the included studies did not report any data on the bioaccessibility and bioavailability of selenium in diet or supplements. Future studies need to be focused on these issues.

The subgroup analysis revealed that high selenium intake was significantly associated with low risk of postpartum depression. Due to the placental transfer of selenium to the fetus, maternal serum selenium levels are reduced during pregnancy, especially in the 3^rd^ trimester. In addition, selenium is secreted in maternal breast milk as a component of selenoproteins. These processes increase the daily selenium requirement of pregnant and lactating women, which may result in selenium deficiency if not compensated properly^55^. It should be noted that supplementary selenium is more effective than dietary selenium in the improvement of low serum selenium levels^56^. In this meta-analysis, all studies conducted on postpartum depression considered supplementary, but not dietary, intake of selenium. This could contribute to the observed significant association between selenium intake and the risk of postpartum depression.

Interestingly, the present meta-analysis indicated that selenium supplementation significantly decreased depressive symptoms. Several mechanisms can explain this beneficial effect of selenium on depression. Selenium is known as a key regulatory factor of inflammatory and oxidative responses. Selenium deficiency can disrupt the function of multiple antioxidant enzymes such as glutathione peroxidase and thioredoxin reductases, which protect cells against oxidative damage^57,58^. Furthermore, inflammation is regarded as a part of depression pathogenesis^59^. Therefore, anti-inflammatory properties of selenium may help to improve depressive symptoms^60,61^. It is also possible that selenium affects depression symptoms through the modulation of neurotransmitter turnover as well as regulation of thyroid function^62,63^.

Several techniques have been suggested to determine serum concentration of selenium including atomic absorption spectrometry, molecular, atomic fluorescence spectrometry, inductively coupled plasma-mass spectrometry (ICP-MS) and graphite furnace atomic absorption spectrometry, flame atomic absorption, electrothermal atomic absorption spectrometry^64–67^. Atomic fluorescence spectrometry has higher sensitivity and is simpler than atomic absorption spectrometry. However, it has some detection limits^68^. Graphite furnace atomic absorption spectrometry is a selective, sensitive and easy method, however it is a single element technique^69,70^. Electrothermal atomic absorption spectrometry requires a small sample volume. This method is sophisticated and expensive^66,71^. Flame atomic absorption spectroscopy as a precise method, requires high sample preparation^72^. Serum or plasma selenium is assessed usually by two common methods of ICP-MS and atomic absorption spectrometry. ICP-MS is higher sensitivity than atomic spectrometry. It has multi-element capability, good stability and detects qualitative and quantitative trace element. However, this method is relatively expensive^73^. Atomic absorption spectrometry has a low detection limit. Although, this method is comparatively inexpensive, it is not an exclusive detection technique^72^. As a result, the different methodologies used to measure selenium in serum may be considered as one of the sources of heterogeneity. The method used to measure serum concentration of selenium in included studies are reported in Supplementary Table 1. Unfortunately, we could not evaluate the effect of this factor on the study findings. It has been proposed that serum concentration of selenium may be affected by sex and age^74^. The association between serum selenium level and gender is not clear. Some previous investigations indicated that there was no significant difference in serum selenium between males and females^75–77^. However, several studies reported serum concentration of selenium was related to gender^78,79^. Some studies reported that serum selenium concentration was higher in men compared with women^78,79^. In contrast, one study revealed that women had higher serum selenium in comparison with men^80^. It is possible that some factors including differences in sexual hormones, smoking and dietary habits play a role in relationship between gender to serum selenium level^80–82^. Also, the findings of studies regarding the effect of age on serum selenium concentration are inconsistent. According to the previous studies, no significant association between serum selenium and age was found^79,83^. However, this finding was not approved by some studies^84,85^. It seems that changes in body selenium distribution, dietary habits and hormonal status probably affect selenium concentration through different ages^82,86–88^. For example, plasma estrogen is positively related to serum selenium. Therefore, change of estrogen status throughout the life cycle can influence serum selenium in women^89^. Moreover, a significant reduction in serum selenium has been reported in elderly individuals^78^. Accumulation of inflammatory factors, change in physiology conditions, inadequate intake of selenium-rich sources and inefficient absorption of dietary selenium are contributed in the relationship between the declined serum selenium level and aging^90–92^.

This study has several limitations. First, there were high levels of heterogeneity in all analyses except for the effect of selenium supplementation on depression scores. Second, due to the insufficient number of studies, we could not run subgroup analyses for all outcomes except for selenium intake and the relative risk of depression. Third, potential confounding factors were not adjusted in some of the included studies, which might affect the findings.

Strengths of the present study should also be considered. First, this study is the first meta-analysis that investigated the role of selenium in depressive disorders. Second, we conducted a comprehensive search using several databases to identify eligible studies. Third, we included both interventional and observational studies in this systematic review and meta-analysis to perform a comprehensive assessment regarding selenium and depression.

## Conclusion

In conclusion, the findings of this systematic review and meta-analysis suggest that high selenium intake may have a protective role against postpartum depression. In addition, our findings support that supplementation with selenium can be effective in reducing depressive symptoms. Nevertheless, further studies are needed to draw definitive conclusions.

## Supplementary Information

Supplementary Table S1.

## Data Availability

The datasets generated during and/or analyzed during the current study are available from the corresponding author on reasonable request.

## Abbreviations

NOS: The Newcastle–Ottawa Scale
CCRBT: The Cochrane Collaboration Risk of Bias Tool
SE: Standard errors
SD: Standard deviations
CI: Confidence interval
WMD: Weighted mean difference
OR: Odds ratio
r: Correlation
