# Supplementary material for: The role of selenium in depression: a systematic review and meta-analysis of human observational and interventional studies
Source: Sci Rep. 2022 Jan 20;12:1045. doi: 10.1038/s41598-022-05078-1 (PMC8776795; doi:10.1038/s41598-022-05078-1)
Supplement: Supplementary file 1 — Supplementary Table S1. [file 41598_2022_5078_MOESM1_ESM.docx]

**Supplementary Table 1**: Serum concentration, dietary or supplementary intake of selenium, and method used to measure dietary/serum selenium

| **Study** | **Measurement** | **Serum concentration /dietary or supplementary intake of selenium** | **Method** |
| --- | --- | --- | --- |
| Amini et al. | Dietary selenium | Mean (mg/d):  Cases: 6.5  Controls: 23.7 | FFQ |
| Banikazemi et al. | Dietary selenium | NR | 24-h dietary recall |
| Conner et al. | Serum selenium | Mean±SD (𝞵g/L):  82±18  (49 to 450) | ICP-MS |
| Ekramzadeh et al. | Serum selenium | Mean±SD (𝞵g/L):  Case:94.78±10.80  Control: 111.007±15.93 | GFAAS |
| Ghimire et al. | Serum selenium | Median (IQR) (𝞵g/L):  Cases: 192.2 (176.9_208.9)  Controls: 194.1 (179.7_209.3) | ICP-MS |
|  | Dietary selenium | Quintiles: (𝞵g/d)  Q1: ≤72.5  Q2: >72.5 to ≤95.5  Q3: >95.5 to ≤118.1  Q4: >118.1 to ≤149.8  Q5: >149.8 | 24-h dietary recall |
| Gosney et al. | Serum selenium | Mean: (𝞵M/L):  Baseline :1.03  After intervention: 1.1 | ICP-MS |
| Ibarra et al. | Serum selenium | Mean±SD (𝞵g/L):  Diet rich in selenium:  Baseline: 85.89±18.9  After intervention: 90.5±37.6  Control diet:  Baseline: 86.23±15.8  After intervention: 88.75±34.5 | NR |
| Islam et al. | Serum selenium | Mean±SE (mg/L):  Cases: 0.03±0.002  Controls: 0.07±0.003 | FAAS  GFAAS |
| Jin et al. | Plasma selenium | Tertiles (𝞵g/L):  T1: 92.8 ±3.7  T2: 106 ±3.5  T3: 123.7 ±14.5 | ICP-MS |
| Leung et al. | Supplementary selenium intake | Mean±SE (Mg/d):  Cases: 19±13  Controls: 25±17 | The Supplement Intake Questionnaire |
| Li et al. | Dietary selenium | Median ( IQR) (𝞵g/d):  Cases: 97.45(67-30)  Controls: 115.00(73-60) | 24-h recall |
| Mokhber et al. | Serum selenium | Mean±SE (𝞵g/dl):  Selenium supplementation:  Baseline: 122.5±23.2  After intervention: 168.6±36.4 | Electrothermal atomic  absorption spectrometry |
|  |  | Placebo:  Baseline:122.9 ±26.9  After Intervention: 119.4±33.4 |  |
| Pasco et al. | Dietary selenium | Median ( IQR) (𝞵g/d):  71.2 (55.9-87.0) | Semi-quantitative FFQ |
| Perez-Cornago et al. | Dietary selenium | Mean±SE (𝞵g/d) :  Cases: 116.3±5.3  Controls: 128.9±5.3 | 48-hour weighted food record |
| Samad et al. | Serum selenium | Mean (ng/mL):  Cases: 60  Controls: 98 | FAAS |
| Sánchez‑Villegas et al. | Dietary selenium | NR | Semi-quantitative  FFQ |
| Shor–Posner et al. | Serum selenium | Mean±SD (𝞵g/L):  Selenium supplementation:  Baseline:123±25  After Intervention: 123.5±23  Placebo  Baseline: 119±20  After Intervention:108.7±15 | standard fluorometric |
| Singh et al. | Dietary selenium | Mean±SD (𝞵g/d):  94.5±60.2 | ASA24-hour dietary recall |
| Wieder-Huszla et al. | Serum selenium | Mean±SD (mg dm^-3^):  0.06±0.03 | absorption spectrometry |
| Tatt et al. | Dietary selenium | Mean±SD (𝞵g/d):  Total: 81.9±54.44 | 24-hour dietary recall |

FAAS: flame atomic absorption spectrophotometry; FFQ: food-frequency questionnaire; ASA24: Automated Self-Administered 24-hour Dietary Recall; (GFAAS): graphite furnace atomic absorption spectrometry; SD: standard deviation; SE: standard error; ICP-MS: inductively coupled plasma-mass spectrometry; NR: not reported
